# How much is the lack of retention evidence costing trial teams in Ireland and the UK?

**DOI:** 10.1186/s13063-022-06223-x

**Published:** 2022-05-12

**Authors:** Ellen Murphy, Frances Shiely, Shaun Treweek

**Affiliations:** 1 Trials Research and Methodologies Unit (TRAMS), Health Research Board Clinical Research Facility at University College Cork, Cork, Ireland; 2 School of Public Health, University College Cork, Cork, Ireland; 3 Health Services Research Unit, University of Aberdeen, Aberdeen, UK

**Keywords:** Randomised trials, Trial retention, Retention strategies, Cost

## Abstract

**Background:**

Evidence to support the use of many retention strategies in clinical trials is lacking. Despite this, trial teams still need to have some form of retention strategy in their trials to try and avoid high attrition rates. This study aimed to estimate how much this lack of retention evidence might be costing trials in Ireland and the UK.

**Methods:**

We selected the top ten most routinely used retention strategies by Clinical Trial Units in the UK and made assumptions as to how each of these strategies was most likely to be implemented and the costs involved in doing this. We applied our costing model to a hypothetical trial scenario in both Ireland and the UK as well as to three published trial protocols. We developed the costing model and calculated the costs in Microsoft Excel.

**Results:**

Retention strategies were often poorly specified, meaning we had to make assumptions about implementation and in some cases about the strategy itself. Based on our assumptions, some retention strategies can be extremely expensive; some of the costliest strategies included “data collection scheduled with routine care” (€900–€32,503.25), “a timeline of participant visits for sites”—with integrated participant reminder (€304.74–€14,803.70), and “routine site visits by CTU staff” and “investigator meetings face to face”, both costing (€777.67–€14,753.48). Others such as “telephone reminders for questionnaire response” (€34.58–€568.62), “a timeline of participant visits for sites”—site reminder alone (€79.18–€112.23), and “targeted recruitment of sites/GPs” (€30–€1620) were less costly compared to the other strategies.

**Discussion:**

The resources invested in the use of some retention strategies may outweigh known or imagined benefits on retention. Where benefits are currently unknown, evaluation should be a priority.

**Conclusion:**

More evaluation of the effectiveness and cost of trial retention strategies is needed to avoid widespread use of strategies that are both expensive and ineffective.

**Supplementary Information:**

The online version contains supplementary material available at 10.1186/s13063-022-06223-x.

## Background

Randomised trials can be no better than the data they collect. If retention has been poor, in other words, data are missing for many participants, then the usefulness of the trial starts to come into question. Patchy datasets can lead to a trial becoming underpowered for its primary outcome and very underpowered for its secondaries, which were anyway probably not part of power calculations. Participants who do not provide data may differ to those who remain [1–3], making interpretation harder. It might be possible to overturn trial conclusions by simply imagining that the results from missing participants had gone against those conclusions [4]. In short, potential users of the trial results now have doubts and doubt undermines trials.

Poor retention is a major cause of research waste as it can delay the implementation (or removal) of healthcare interventions [5], as missing primary outcome data can reduce the power of the study to detect significant findings [6]. Poor retention may also increase trials costs [5, 7]. A recent prioritisation exercise (PRioRiTy 2) identified 20 priority unanswered research questions for research in trial retention, including questions around what motivates participants to stay involved, how to provide information and how what is done at recruitment might influence retention [8].

Although there are plenty of unanswered research questions in trial retention, trial teams nevertheless have to use some form of retention strategy in their trials. In the UK, Clinical Trials Units (CTUs) registered with the UK Clinical Research Network use many approaches and Kearney et al. asked them what these strategies were. Thirty-three (70%) CTUs responded outlining commonly used strategies [6]. While evidence is available for some strategies, none have compelling evidence of benefit [9].

The current study aimed to estimate how much this lack of retention evidence might be costing trials in the UK and Ireland. When referring to retention strategy we mean an activity that is done with the explicit aim of improving trial data completeness and/or quality. Trialists have many options for how to do this but we focus on estimating the costs of the ten retention strategies used most often by UK CTUs as identified in the study by Kearney and colleagues [6].

## Methods

The top ten most routinely used retention strategies used by UK CTUs are shown in Table 1, along with the percentage of CTUs that use this strategy routinely [6] and the evidence of benefit provided by the most recent Cochrane Systematic Review [9]. The top ten strategies were identified in a survey of UK CTUs [6], where respondents outlined the strategies they use to improve retention. However, the responses provided to Kearney and colleagues did not contain detail as to how trial teams implement these strategies. We extracted as much information as possible from the paper and Dr Kearney provided additional information where available but we were nevertheless left to make many assumptions as to how retention strategies were implemented, particularly for the strategy “a timeline of participant visits”, where the very nature of the strategy itself remained unclear.

**Table 1:** Top 10 most used missing data strategies by CTUs in the UK [6]

	Missing data strategies	Number (N) and percentage of CTUs that routinely use strategies	Evidence of reported evaluations into their effectiveness according to the most recent Cochrane Systematic Review [9].
**1**	Newsletters	*N*=23, 70%	“The evidence is very uncertain about the effect on retention of including a newsletter compared to no newsletter; RD = − 0% (95%CI, − 4% to 3%); GRADE; very low”, evidence is based on four studies from various disciplines (*n*=5622)
**2**	A timeline of participant visits for sites	*N*=19, 58%	No evidence
**3**	Inclusion of prepaid envelopes (questionnaires)	*N*=19, 58%	“Various strategies compared to usual practice for return postage, such as free post versus second class stamp, high priority mail stamp versus usual postage and personal form may increase retention slightly: RD= 4% (95% CI − 0% to 9%), GRADE; low”. Evidence is based on three studies, (*n*=1543)
**4**	Telephone reminders	*N*=18, 55%	“Telephone reminders compared to postal reminders may result in a large increase in retention, evidence is from one study [10] (RD= − 19% (95%CI − 33% to − 5%) GRADE low (− 1 level: study limitations unclear risk of bias; − 1 level: imprecision single study, *n*=148)” “Telephone reminders compared to usual follow-up^a^ may result in little or no difference in retention, (smoking cessation [11] (RD = − 1% (95% − 18% to 15%) GRADE low, (− 2 levels: imprecision-single study, *n* = 127; wide CI crossing RD = 0))” ^b^
**5**	Data collection scheduled with routine care	*N*=18, 55%	No evidence
**6**	Site initiation training on missing data	*N*=18, 55%	No evidence
**7**	Investigator meetings face to face	*N*=17, 52%	No evidence
**8**	Routine site visits by CTU staff	*N*=15, 45%	No evidence
**9**	Targeted recruitment of sites/GPs	*N*=15, 45%	No evidence
**10**	Flexibility in appointment times	*N*=15, 45%	No evidence

GRADE, grades of evidence; low certainty: the confidence in the effect estimate is limited; the true effect may be substantially different from the estimated effect. Very low certainty: very little confidence in the effect estimate; the true effect is likely to be substantially different from the estimate of the effect [9]

^a^From this single study “usual follow-up” is as follows; “We followed-up participants by any of the means they agreed to at the start of the trial, including post, e-mail, and telephone calls to mobile, home, or work numbers [12]. We used all the effective evidence-based methods that were feasible to introduce into the procedures of the trial [12], as identified in the systematic reviews by Edwards *et al*. and Hoile et al. [13]. These included monetary incentives, posting correspondence by recorded delivery, pre-notification, follow-up contact, unconditional advance cash incentives, short, concise questionnaires, duplicate questionnaires sent at repeat follow-up attempts, mentioning that commitment to the trial implied an obligation to respond, mention of university sponsorship, prepaid return envelopes with stamps, an assurance of confidentiality, and first-class outward mailing” [11]

^b^We present all evidence regarding telephone reminders however we chose to select telephone reminders compared to the usual follow-up in Table 4 to calculate the cost per participant retained as we believe it is the most relevant comparison for trial teams

To estimate the cost of the retention strategies, we made assumptions as to how each of these strategies would be implemented and the costs involved. We called this our costing model for each strategy. We contacted experienced clinical trial professionals such as trial managers, clinical research nurses, and professionals working in clinical research facilities in the UK and Ireland for information to inform our costing models. We identified these individuals through workplace inquiries and personal knowledge of suitable personnel to answer specific costing queries.

We applied our costing models to each of the ten strategies in two ways. First, we created a hypothetical trial scenario (Table 2). We made assumptions about how the retention strategies would most likely be conducted and then calculated and applied the costs of running each of the ten retention strategies in both Ireland and the UK. The costing models for some strategies differed slightly between Ireland and the UK due to differences in the responsibilities held by staff members involved in running clinical trials and differences in how some retention strategies are conducted in each country (See Additional file 1 for full details).

**Table 2:** Hypothetical trial characteristics

Trial characteristics	
**Number of participants**	500
**Number of sites**	10 sites
**Duration of participant follow-up**	1 year
**Number of trial visits**	3
**Data collection**	1 questionnaire is sent out: three trial visits
**Location of the trial**	Ireland/UK

Secondly, we chose three trial protocols published in the journal *Trials* between 2016 and 2020. We chose these three for convenience reasons: they fit into our costing model well, represented trials of various sizes, and the trials had different characteristics. We used these to estimate the cost associated with each retention strategy in a “real-life” randomised controlled trial. We applied our costing model for the previously identified top ten retention strategies to each of the three randomised controlled trials regardless of whether the trial protocol stated that these retention methods were used or not.

An example costing model for both a hypothetical trial and published trial protocol [14], with costs and assumptions, is shown in Table 3. A full description of the costing models and the assumptions we made to create them, together with details of the three trials selected from the *Trials* journal are outlined in Additional file 1*.*

**Table 3:** Assumptions and costings for “Newsletters”—hypothetical trial Ireland and the MAMI trial [14]

Strategy 1	Assumptions made
**Hypothetical trial (Ireland)**	
**Newsletter**	→ 2 newsletters sent out over the 1-year trial period. → 5 h to develop the newsletter. → 2 h work to electronically send out 500 newsletters. → 8 h to manually post out 500 newsletters. → Stamp costs in Ireland = €1.10. → Mailchimp subscription is €151.78 for 1 year. → Developing the newsletter carried out by a Research Nurse (€54 hourly rate) → Emailing/posting out the newsletter carried out by a Research Assistant (€25 hourly rate)
**Costings**	**Costings** **Staff costs for developing and manually posting out the newsletter:** → Developing newsletter: 5 h × €54 = €270. → Manually posting newsletter: 8 h × €25 = €200 → Total staff costs = €470 **Postage (stamp) costs:** → 500 × €1.10 = €550 **Total costs: Staff hours plus postage costs × frequency of activity:** → €1020 × 2 = **€2040** **Staff costs for developing and electronically posting out the newsletter twice a year:** → Developing newsletter: 5 h × €54 = €270. → Electronically posting newsletter: 2 h × €25 = €50. → Total staff costs = €320. **Electronic postage costs:** Mailchimp = €151.78 **Total costs: Staff costs × frequency of activity plus flat rate of Mailchimp:** → €320 × 2 = €640 plus €151.78= **€791.78**
**MAMI trial (Trials Journal)**	
**Newsletter**	→ 4 newsletters sent out over the 2-year trial period → 5 h to develop the newsletter. → 0.24 h to electronically send out 60 newsletters (2 h work to electronically send out 500 newsletters, therefore 60 newsletters is 0.24 h) → 0.96 h of work to manually stuff and post 60 newsletters (8 h to manually post out 500 newsletters, therefore 60 newsletters take 0.96 h) → Stamp costs in the Netherlands = €0.96 → Mailchimp subscription for two years is €303.56 (€151.78 for 1 year) → Developing the newsletter carried out by a Research Nurse (€30 hourly) → Emailing/posting out the newsletter carried out by a Research Assistant (€17 hourly)
**Costings**	**Costings** **Staff costs for developing and manually posting out the newsletter twice a year:** → Developing newsletter: 5 h × €30 = €150 → Manually posting newsletter: 0.96 h × €17 = €16.32 → Total staff costs = €166.32 **Postage (stamp) costs:** → 60 × €0.96 = €57.60 **Total costs: Staff hours plus postage costs × frequency of activity:** → €223.92 × 4 = **€895.68** **Staff costs for developing and electronically posting out the newsletter twice a year:** → Developing newsletter: 5 h × €30 = €150 → Electronically posting newsletter: 0.24 h × €17 = €4.08 → Total staff costs = €154.08 **Electronic postage costs:** Mailchimp = €151.78 × 2 = €303.56 **Total costs: Staff costs × frequency of activity plus flat rate of Mailchimp:** €154.08 × 4 = €616.32 plus €303.56= **€919.88**

In addition to the ten most routinely used retention strategies, we further elaborated and included additional actions that are likely to be carried out by trial units when trying to retain participants that were not specifically outlined by Kearney et al. [6]. These assumptions reflect actions that are likely to be conducted by trial teams during the implementation of the listed strategies. For example, under the strategy “inclusion of pre-paid envelopes (questionnaires)” along with sending out pre-paid envelopes to enhance questionnaire response we also assumed a reminder schedule would be sent out to 30% of participants who initially did not return the questionnaire. A full list of the additional assumptions can be found in Additional file 1.

Where there was evidence of effectiveness of the retention strategy we calculated the cost per participant retained. For example for the ease of calculation, we calculated the cost per participant retained by using pre-paid return envelopes based on a 4% benefit [9]. In the Irish hypothetical trial scenario, 4% of 500 participants is 20. The total cost of “inclusion of pre-paid envelopes (questionnaires)” was €1690. Therefore the cost per participant retained was €84.50.

All our calculations were done within Microsoft Excel. Additional file 2 contains our Excel spreadsheet with our cost calculations. For ease of comparison, all our costs are presented in Table 4 in both EUR and GBP based on exchange rates of 1 GBP = 1.16279 EUR, 1 EUR = 0.860001 GBP, 1 USD = 0.843802 EUR and 1 USD = 0.725647 GBP, taken from Xe Currency Converter - LIVE Foreign Exchange Rates on the 7th of September 2021.

**Table 4:** Total cost and cost per participant for each retention strategy for all trials

Strategy	Cost				CINNAMON trial [16]
	Ireland (Hypothetical trial)	UK (Hypothetical trial)	MAMI trial [14]	MOON trial [15]	
**Strategy 1: Newsletters (posted)**	€2040 (£1754.40)	€1657.67 (£1425.60)	€895.68 (£770.29)	€755.17 (£649.45)	€1931.83 (£1661.33) ($2289.44)
**Assumptions**	2 newsletters per year; 500 participants; 1-year trial period.	2 newsletters per year; 500 participants; 1-year trial period.	2 newsletters per year; *60 participants; 2-year trial period*^a^*.*	2 newsletters per year; *154 participants; 1-year trial period.*	2 newsletters per year; *428 participants; 2-year trial period.*
**Effect estimate**^b^	“The evidence is very uncertain about the effect on retention of including a newsletter compared to no newsletter: RD = − 0% (95% CI − 4% to 3%); GRADE: very low”				
**Cost per participant retained**	Current evidence suggests no retention benefit, unable to calculate cost per participant retained due to lack of evidence				
**Strategy 1: Newsletters (emailed)**	€791.78 (£680.93)	€639.45 (£549.93)	€919.88 (£791.10)	€546.59 (£470.07)	€1094.17 (£940.96) ($1296.72)
**Assumptions**	2 newsletters electronically sent per year; 500 participants; 1 year trial period	2 newsletters electronically sent per year; 500 participants; 1 year trial period	2 newsletters electronically sent per year; *60 participants; 2-year trial period*	2 newsletters electronically sent per year; *154 participants; 1 year trial period*	2 newsletters electronically sent per year; *428 participants; 2-year trial period*
**Effect estimate**	“The evidence is very uncertain about the effect on retention of including a newsletter compared to no newsletter: RD = − 0% (95% CI − 4% to 3%); GRADE: very low”				
**Cost per participant retained**	Current evidence suggests no retention benefit, unable to calculate cost per participant retained due to lack of evidence				
**Strategy 2: A timeline of participant visits for sites (site reminder)**^c^	€79.18 (£68.09)	€111.95 (£96.28)	€101.34 (£87.15)	€111.95 (£96.28)	€112.23 (£96.52) ($133.01)
**Assumptions**	Data Manager develops reminder schedule software, electronically emails the software to the sites (10 sites). Carried out once. This software is used to notify staff of upcoming participant trial visits	Data Manager develops reminder schedule software, electronically emails the software to the sites (10 sites). Carried out once. This software is used to notify staff of upcoming participant trial visits	Data Manager develops reminder schedule software, electronically emails the software to the sites *(1 site).* Carried out once. This software is used to notify staff of upcoming participant trial visits	Data Manager develops reminder schedule software, electronically emails the software to the sites *(1 site).* Carried out once. This software is used to notify staff of upcoming participant trial visits	Data Manager develops reminder schedule software, electronically emails the software to the sites *(5 sites).* Carried out once. This software is used to notify staff of upcoming participant trial visits
**Effect estimate**	No evidence provided in the systematic review				
**Cost per participant retained**	Unable to calculate cost per participant retained due to lack of evidence				
**A timeline of participant visits for sites (participant reminder)**^c^	€9232.18 (£7939.68)	€6103.57 (£5249.08)	€304.74 (£262.08)	€1957.37 (£1683.34)	€14803.70 (£12730.79) ($17544.05)
**Assumptions**	Research nurse contacts 500 participants – 5 min per call-carried out once for each visit (3 visits) **Failed contact attempts** – research nurse contacts 500 participants - 2 failed contact attempts (1 min each) – carried out once for each visit (3 visits)	Trial Manager contacts 500 participants – 5 min per call-carried out once for each visit (3 visits) **Failed contact attempts** – trial manager contacts 500 participants - 2 failed contact attempts (1 min each) – carried out once for each visit (3 visits)	Research nurse contacts *60 participants* – 5 min per call-carried out once (1 trial visit) **Failed contact attempts** – research nurse contacts *60 participants* - 2 failed contact attempts (1 min each) – carried out once (1 trial visit)	Trial Manager contacts *154 participants* – 5 min per call-carried out once for each visit (*3 visits*) **Failed contact attempts** – trial manager contacts *154 participants* - 2 failed contact attempts (1 min each) – carried out once for each visit *(3 visits)*	Research nurse contacts *428 participants* – 5 min per call-carried out once for each visit (*9 trial visits*) **Failed contact attempts** – research nurse contacts *428 participants* - 2 failed contact attempts (1 min each) – carried out once for each visit *(9 trial visit)*
**Effect estimate**	No evidence provided in the systematic review				
**Cost per participant retained**	Unable to calculate cost per participant retained due to lack of evidence				
**Strategy 3: Inclusion of pre-paid envelopes (questionnaire)**	€1690.00 (£1453.40)	€1346.56 (£1158.04)	€170.98 (£147.04)	€832.79 (£716.20)	€667.40 (£573.94) ($790.94)
**Assumptions**	1 questionnaire to all 500 participants; cost of stamp to send out and to return; 1 reminder to 30% of participants (150); cost of stamp to send out and to return;	1 questionnaire to all 500 participants; cost of stamp to send out and to return; 1 reminder to 30% of participants (150); cost of stamp to send out and to return	*1 questionnaire to all 60 participants*; cost of stamp to send out and to return; 1 reminder to 30% of participants (18); cost of stamp to send out and to return	*2 questionnaires to all 154 participants*; cost of stamp to send out and to return; 1 reminder for each questionnaire to 30% of participants (47); cost of stamp to send out and to return	1 questionnaire to all *428 participants*; cost of stamp to send out and to return; 1 reminder to 30% of participants (129); cost of stamp to send out and to return
**Effect estimate**	“Various strategies compared to usual practice for return postage, such as free post versus second class stamp; high priority mail stamp versus usual postage; and personal form may increase retention slightly: RD = 4% (95% CI − 0% to 9%); GRADE: low” ^d^				
**Additional participants retained**	20	20	3	7	18
**Cost per participant retained**	€84.50 (£72.67)	€67.33 (£57.90)	€57 (£s49.02)	€118.97 (£102.31)	€37.08 (£31.88) ($43.94)
**Strategy 4: Telephone reminders**					
**Telephone reminders for trial visits**	€9153 (£7871.59)	€5991.62 (£5152.80)	€203.40 (£174.92)	€1845.42 (£1587.06)	€14691.47 (£12634.27) ($17411.04)
**Assumptions**	Research nurse contacts 500 participants– 5 min per call-carried out once for each visit (3 visits) **Failed contact attempts** – research nurse contacts 500 participants - 2 failed contact attempts (1 min each) – carried out for each visit (3 visits)	Trial Manager contacts 500 participants– 5 min per call-carried out once for each visit (3 visits) **Failed contact attempts** – trial manager contacts 500 participants - 2 failed contact attempts (1 min each) – carried out for each visit (3 visits)	Research nurse contacts *60 participants*– 5 min per call-carried out once (1 trial visit) **Failed contact attempts** – research nurse contacts *60 participants* - 2 failed contact attempts (1 min each) – carried out once (1 trial visit)	Trial Manager contacts *154 participants*– 5 min per call-carried out once for each visit (*3 visits*) **Failed contact attempts** – trial manager contacts *154 participants* - 2 failed contact attempts (1 min each) – carried out for each visit *(3 visits)*	Research nurse contacts *428 participants*– 5 min per call-carried out once for each visit (*9 trial visits*) **Failed contact attempts** – research nurse contacts *428 participants* - 2 failed contact attempts (1 min each) – carried out once for each visit *(9 trial visit)*
**Effect estimate**	“Telephone reminders compared to usual follow-up may result in little or no difference to retention (smoking cessation (14)): RD = − 1% (95% CI -18% to 15%); GRADE low (− 2 levels: imprecision-single study, *n* = 127; wide CI crossing RD = 0)” ^e^				
*Cost per participant retained*	With a risk difference of negative 1 there are no participants retained with this strategy so cost per participant retained cannot be calculated				
**Telephone reminders for questionnaire response**	€423.75 (£364.43)	€568.62 (£489.01)	€34.58 (£29.74)	€356.33 (£306.44)	€246.00 (£211.56) ($291.54)
**Assumptions**	Research assistant contacts 30% of participants (150) – 5 min per call – carried out once **Failed contact attempts** – research assistant contacts 150 participants– 2 failed attempts - 1 min per attempt –carried out once	Research assistant contacts 30% of participants (150) – 5 min per call – carried out once **Failed contact attempts** – research assistant contacts 150 participants– 2 failed attempts - 1 min per attempt –carried out once	Research assistant contacts 30% of participants (18) – 5 min per call – carried out once **Failed contact attempts** – research assistant contacts 18 participants– 2 failed attempts - 1 min per attempt –carried out once	Research assistant contacts 30% of participants (47)– 5 min per call – carried out twice **Failed contact attempts** – research assistant contacts 47 participants– 2 failed attempts - 1 min per attempt –carried out twice	Research assistant contacts 30% of participants (129) – 5 min per call – carried out once **Failed contact attempts** – research assistant contacts 129 participants– 2 failed attempts - 1 min per attempt –carried out once
**Effect estimate**	“Telephone reminders compared to usual follow-up may result in little or no difference to retention (smoking cessation [11]): RD = − 1% (95% CI −18% to 15%); GRADE low (− 2 levels: imprecision-single study, *n* = 127; wide CI crossing RD = 0)”^e^				
*Cost per participant retained*	With a risk difference of negative 1 there are no participants retained with this strategy so cost per participant retained cannot be calculated				
**Telephone reminders for at home data collection**			€244.08 (£209.91)		
**Assumptions**			Research nurse contacts 30% of participants [18] – 5 min per call - 1 reminder for each at *home data collection (4 in total)*		
**Effect estimate**	“Telephone reminders compared to usual follow-up may result in little or no difference to retention (smoking cessation [11]): RD = − 1% (95% CI − 18% to 15%); GRADE low (− 2 levels: imprecision-single study, *n* = 127; wide CI crossing RD = 0)”^e^				
*Cost per participant retained*	With a risk difference of negative 1 there are no participants retained with this strategy so cost per participant retained cannot be calculated				
**Strategy 5: Data collection scheduled with routine care**	€20250 (£17415.02)	€15697.67 (£13500)	€900 (£774.00)	€4834.88 (£4158)	€32503.25 (£27951.92) ($38520)
**Assumptions**	Carried out by a research nurse during a routine care visit – 15 min – carried out 3 times for each participant (once for each trial visit)	Carried out by a research nurse during a routine care visit – 15 min – carried out 3 times for each participant (once for each trial visit)	Carried out by a research nurse during a routine care visit – 15 min – carried out twice for each participant (*outpatient clinic data collection and data collection at birth*)	Carried out by a research nurse during a routine care visit – 15 min – carried out 3 times for each participant - once for each trial visit *(3 trial visits)*	Carried out by a research nurse during a routine care visit – 15 min – carried out 9 times for each participant - once for each trial visit *(9 trial visits)*
**Effect estimate**	No evidence provided in the systematic review				
*Cost per participant retained*	Unable to calculate cost per participant retained due to lack of evidence				
**Strategy 6: Site initiation training on missing data**	€7000 (£6020.00)	€7376.74 (£6344)	€400 (£344.00)	€388.83 (£334.40)	€2953.31 (£2539.76) ($3500)
**Assumptions**	Prep, site training and report per site @€400 per day plus travel and overnight costs. Carried out once for each site (10 sites)	Prep, site training and report by Trial manager (11 h of work) – plus travel and overnight costs. Carried out once for each site (10 sites)	Prep, site training and report per site @€400 per day. Carried out once *(1 site)*	Prep, site training and report by Trial Manager (11 h of work) Carried out once *(1 site)*	Prep, site training and report per site @€400 per day plus travel and overnight costs. Carried out once for each site *(5 sites)*
**Effect estimate**	No evidence provided in the systematic review				
*Cost per participant retained*	Unable to calculate cost per participant retained due to lack of evidence				
**Strategy 7: Investigator meetings face to face**	€14000 (£12040.01)	€14753.48 (£12688)	€1600 (£1376)	€777.67 (£668.80)	€11813.22 (£10159.06) ($14000)
**Assumptions**	Prep, meeting, and report per site @€400 per day plus travel and overnight costs. Carried out twice per site over 1 year trial period (10 sites)	Prep, meeting, and report by Trial manager (11 h of work) – plus travel and overnight costs. Carried out twice per site over 1 year trial period (10 sites)	Prep, meeting, and report per site @€400 per day Carried out 4 times for each site over 2-year trial period *(1 site)*	Prep, meeting, and report by Trial manager (11 h of work). Carried out twice per site over 1 year trial period *(1 site)*	Prep, meeting, and report per site @€400 per day plus travel and overnight costs. Carried out 4 times for each site over 2-year trial period *(5 sites)*
**Effect estimate**	No evidence provided in the systematic review				
*Cost per participant retained*	Unable to calculate cost per participant retained due to lack of evidence				
**Strategy 8: Routine site visits by CTU staff**	€14000 (£12040.01)	€14753.48 (£12688)	€1600 (£1376)	€777.67 (£668.80)	€11813.22 (£10159.06) ($14000)
**Assumptions**	Prep, site visit and report per site @€400 per day plus travel and overnight costs. Carried out twice per site over 1 year trial period (10 sites)	Prep, site visit and report by Trial manager (11 h of work) – plus travel and overnight costs. Carried out twice per site over 1 year trial period (10 sites)	Prep, site visit and report per site @€400 per day Carried out 4 times per site over 2-year trial period *(1 site)*	Prep, site visit and report by Trial manager (11 h of work). Carried out twice per site over 1 year trial period *(1 site)*	Prep, site visit and report per site @€400 per day plus travel and overnight costs Carried out 4 times per site over 2-year trial period *(5 sites)*
**Effect estimate**	No evidence provided in the systematic review				
*Cost per participant retained*	Unable to calculate cost per participant retained due to lack of evidence				
**Strategy 9: Targeted recruitment of sites/GPs**^f^	€1620 (£1393.20)	€1060.46 (£912)	€30 (£25.80)	€35.35 (£30.40)	€506.28 (£435.39) ($600)
**Assumptions**	Research nurse carries out site selection and investigation (1 h work per site) – 30 sites are targeted	Trial manager carries out site selection and investigation (1 h work per site) – 30 sites are targeted	Research nurse carries out site selection and investigation (1 h work per site) –single centre so only 1 site targeted	Trial manager carries out site selection and investigation (1 h work per site) – single centre so only 1 site targeted	Research nurse carries out site selection and investigation (1 h work per site) – 15 sites are targeted
**Effect estimate**	No evidence provided in the systematic review				
*Cost per participant retained*	Unable to calculate cost per participant retained due to lack of evidence				
**Strategy 10: Flexibility in appointments**	€4050 (£3483.00)	€3139.53 (£2700)	€270 (£232.20)	€1004.65 (£864)	€2177.01 (£1872.17) ($2580)
**Assumptions**	Research nurse carries out out-of-hours visits for 10% of participants (50) – 1.5-h visit	Research nurse carries out out-of-hours visits for 10% of participants (50) – 1.5-h visit	Research nurse carries out out-of-hours visits for 10% of participants (6) – 1.5-h visit	Research nurse carries out out-of-hours visits for 10% of participants (16) – 1.5-h visit	Research nurse carries out out-of-hours visits for 10% of participants (43) – 1.5-h visit
**Effect estimate**	No evidence provided in the systematic review				
*Cost per participant retained*	Unable to calculate cost per participant retained due to lack of evidence				

^a^Italic font indicates the real trial information that we used, e.g. number of trial participants, number of trial sites, number of trial visits and any retention methods used within the trials, e.g. MOON trial sent out 2 questionnaires to participants

The non-italic font which indicates the assumptions we applied. A full description of the trial characteristics and any retention activities conducted by the real-life trials are documented in Additional file 1

^b^Evidence from the most recent Cochrane Systematic Review [9]

^c^There was no clear explanation as to what this strategy entails therefore we made the following two assumptions. *Assumptions for “a timeline of participant visits for sites – site reminder”*: The data manager develops reminder schedule software, which is costed as 3 h of work, the software is emailed to the trial sites for the sites to use, costed at 10 min of work. This reminder schedule software can be modified to suit the trial follow-up schedule and to notify staff of upcoming participant trial visits. *Assumption for “a timeline of participant visits for sites – participant reminder”*: this costing assumption includes the cost of the “site reminder” plus the cost of the trial manager/research nurse telephoning each participant for each visit (5 min of work) plus the cost of failed telephone contact attempts (2 attempts per participant)

^d^We assumed benefit was applied across the whole response to all cycles. The Cochrane review found that return postage which included “preaddressed second class stamped envelope”, “high priority stamp to the mailing” and “personalised postal follow-up” all combined likely lead to a 4% benefit, so it is likely that pre-paid envelopes on their own may not provide a 4% benefit on retention. For the ease of calculation, we calculated the cost per participant retained by using pre-paid envelopes based on a 4% benefit

^e^We chose to select telephone reminders compared to usual follow up compared to postal follow up in as we believe it is the most relevant comparison for trial teams

^f^Assumptions for “targeted recruitment of GPs/sites” again there was no clear information what this strategy entails therefore we interpreted this as trial teams targeting sites that would be most likely to participate/sites that had the facilities and experience to participate/sites that have conducted similar trials previously, we costed the time taken to conduct background research into which sites these would be and associated investigation into recruitment

## Results

We discussed the top ten most routinely used trial retention strategies in the UK and made assumptions as to how each of these strategies would be implemented and the costs involved. We applied this costing model to our hypothetical trial scenario, (1-year participant follow-up, 10 sites, 500 participants, 3 trial visits, 1 questionnaire sent out) in Ireland and the UK, and found that the strategies ranged from very cheap to very expensive to implement. The cheapest strategy to implement in both countries would be “a timeline of participant visits for sites”—site reminder alone costing €79.18 (£68.09) and €111.95 (£96.28), respectively, and the most expensive strategy would be “data collection scheduled with routine care” costing €20,250 (£17,415.02) in Ireland and €15,697.67 (£13,500) in the UK.

For the “real life” trials, “a timeline of participant visits for sites”—site reminder alone would be the cheapest strategy in the CINNAMON trial (€112.23 (£96.52)). For the MAMI and MOON trials, “targeted recruitment of sites/GPs” would be the cheapest strategy in these two single-centre trials costing €30 (£25.80) and €35.35 (£30.40), respectively. The most expensive strategy in the MAMI trial would be either “investigator meetings face to face” or “routine site visits by CTU staff” both costing €1600 (£1376). For the MOON and CINNAMON trials, the costliest strategy for the trial teams to use would be “data collection scheduled with routine care” costing €4834.88 (£4158) and €32,503.25 (£27,951.92), respectively.

The cost of retention strategies differed across the trials due to differences in the trial size (number of sites and participants), staff roles and responsibilities in different countries and the number of follow-up visits. For the smaller single-site trials, such as the MAMI trial (60 participants, 1 site, 1 trial visit) it naturally costs less to implement retention strategies compared to the larger trials such as the CINNAMON trial (428 participants, 5 sites and 9 trial visits).

Newsletters were identified as the most routinely used retention strategy by CTUs in the UK [6]. The cost of sending newsletters would be one of the cheaper retention strategy options ranging from €755.17 (£649.45) to €2040 (£1754.40) for manually posting the newsletters, and €546.59 (£470.07) to €1094.17 (£940.96) to electronically send newsletters. However, the most up-to-date evidence suggests there is no retention benefit when using this strategy “RD = − 0% (95% CI − 4% to 3%); GRADE: very low” [9]. This costing model has shown that “data collection scheduled with routine care” is one of the most expensive strategies across all trials (€900 (£774)–€32,503.25 (£27,951.92)).

The retention strategy with the best available evidence is “inclusion of pre-paid envelopes" for questionnaire return. The cost of this retention strategy ranges from €170.98 (£147.04) to €1,690 (£1453.40), one of the cheapest retention strategies trial teams can use. The Cochrane review found that return postage which included “preaddressed second class stamped envelope”, “high priority stamp to the mailing” and “personalised postal follow-up” may lead to a 4% retention benefit “RD= 4% (95% CI − 0% to 9%)”. However, the evidence was based on three low-quality studies (*n*=1543), and the single study of pre-paid return envelopes itself did not find a beneficial effect on retention [9].

## Discussion

Our findings show that the evidence available to support the ten most-used trial retention strategies by CTUs in the UK is weak or lacking entirely but that the cost of using them can be very large.

The most routinely used strategy outlined by Kearney et al. [6] is “newsletters”, this strategy was found to be one of the cheaper retention methods particularly emailing newsletters (€546.59 (£470.07) to €1094.17 (£940.96)). Another cheap strategy across all the trials would be “telephone reminders for questionnaire response” costing between €34.58 (£29.74) and €568.62 (£489.01). We are able to say that these retention strategies would be cheap, but more evidence is needed to show that they are also effective at retaining trial participants.

The second most routinely used retention strategy outlined by Kearney et al., [6] is “a timeline of participant visits for sites”. The site reminder schedule alone would be cheap, costing between €79.18 (£68.09) and €112.23 (£96.52). Integrating a participant reminder schedule would significantly increase the cost (€304.74 (£262.08)–€14,803.70 (£12,730.79)). Similarly “data collection scheduled with routine care” (€900 (£774.00)–€32,503.25 (£27,951.92)), “routine site visits by CTU staff” (€777.67 (£668.80)–€14,753.48 (£12,688)) and “investigator meetings face-to-face” (€777.67 (£668.80)–€14,753.48 (£12,688)), would also be expensive to implement yet none of these have compelling evidence demonstrating that they are effective at retaining trial participants [9]. They may be very effective. The point is that we cannot say with any certainty whether they work or not, and therefore substantial amounts of money and other resources are potentially being invested into strategies that lead to no improvement in retention. We recognise that participant retention may not be the only reason for some of these strategies to be implemented in a trial. For example “routine visits by CTU staff” may also be used for monitoring purposes and the costs may be justified for purposes other than retaining participants. However, it is worth remembering that all these strategies were specifically identified by CTUs as strategies they use to support retention and if a strategy is to be used to improve retention, the jury is out as to whether the majority of the top ten strategies are effective.

Even some of the less costly strategies have limited evidence showing effectiveness and much of the existing evidence is from single studies often with low GRADE ratings [9]. “Telephone reminders for questionnaire response” (€34.58 (£29.74)–€568.62 (£489.01)), “newsletters (emailed)” (€546.59 (£470.07)–€1,094.17 (£940.96)), “targeted recruitment of sites/GPs” (€30 (£25.80)–€1,620 (£1393.20)) and “a timeline of participant visits for sites”—site reminder alone (€79.18 (£68.09)–€112.23 (£96.52)) would be less costly compared to the other strategies but not all have evidence in support of them. A cheap but ineffective strategy is still not something worth using, especially if it takes resources away from other potentially more useful strategies.

Lack of trial process evidence is a sadly enduring problem and despite the paucity of evidence, trial teams need to try and ensure retention is high. Based on our work, a reasonable approach might be to use strategies that are inexpensive when compared to the overall cost of conducting the trial. Building in an evaluation, perhaps a Study Within a Trial (SWAT) [17] would improve decision-certainly for the next trial. For more expensive strategies, we’d suggest only using them in the context of an evaluation. An estimate of the clinical trial cost per participant in the UK’s National Institute for Health Research Health Technology Assessment (NIHR HTA) Programme is £2,987 GBP [18]. Some of that cost is due to retention problems, often because trial teams increasing the recruitment target to compensate for later retention loss. Having a collection of evidence-informed retention strategies just might bring down the average cost per participant, and perhaps reduce some recruitment problems too.

### Strengths and limitations

We acknowledge that a limitation of this study is that we have had to make assumptions to calculate our cost estimates and these may not be truly representative, or the assumptions made may not be accurate depending on how trials are run, especially those outside of Ireland and the UK. Making assumptions was unavoidable due to the lack of information regarding the strategies available from the study by Kearney et al. [6]. We also acknowledge that others may make different assumptions, that there is not a standardised approach to implementing these strategies and that the assumptions affect the costs. However, to help to address this limitation, we have made the costing spreadsheet available as an additional file, which means readers can modify it to suit their own trial and input their own information on how the strategy will be implemented.

One of the strengths of this study is that regardless of the costs, it highlights the lack of evidence for routinely-used trial retention strategies. Even if our estimates are very wrong, no strategy costs nothing and if there is weak or no evidence in support of it, we should pause and consider what we want to do. If trialists go ahead and use the strategy, we think at least some of them should use SWATs or other research designs to investigate the impact of the strategy on retention. The combination of routine use of a strategy to support retention and a lack of evidence that the strategy actually improves retention is a recipe for research waste.

### Recommendations for future research

This paper highlights the need for further research into the effects of trial retention strategies. The cost of some of the strategies that are currently routinely used is significant, and so is the lack of evidence to support their use. We recommend the wider use of SWATs to evaluate the effects of retention strategies used in clinical trials to avoid persistent and widespread research waste. Replication of evaluations will add to the existing evidence to support/not support the use of these strategies.

We also think it would be useful for trial teams to include clear descriptions of their retention strategies and the associated costs in trial publications. The challenges of how to implement a retention strategy based on current descriptions became very clear in this work. Communicating the costs of retention strategies can be helpful to other trial teams to estimate the budgets required for implementing similar strategies. A better idea of costs will allow for better ‘cost-per-participant-retained’ calculations, which in turn will give trial teams another way to compare retention strategies when making choices about which to use in their trial.

## Conclusions

Without evidence regarding the effectiveness of trial retention strategies, trial teams will continue to put substantial amounts of money into strategies that potentially have no beneficial impact on participant retention. More evaluation of the effectiveness and cost of trial retention strategies is needed to avoid the widespread use of strategies that are both expensive and ineffective.

## Supplementary Information

**Additional file 1.**

**Additional file 2.**

## Data Availability

All datasets supporting the conclusions of this article are included within the articles’ additional files.

## Abbreviations

PRioRiTy 2: Prioritising Retention in Randomised Trials study
CTUs: Clinical Trials Units
SWATs: Studies Within a Trial
NIHR HTA: National Institute for Health Research Health Technology Assessment

## References

[1] Dettori JR (2011). Loss to follow-up. Evid Based Spine Care J.

[2] Gul RB, Ali PA (2010). Clinical trials: the challenge of recruitment and retention of participants. J Clin Nurs.

[3] Cooley ME, Sarna L, Brown JK, Williams RD, Chernecky C, Padilla G, Danao LL (2003). Challenges of recruitment and retention in multisite clinical research. Cancer Nurs.

[4] Walsh M, Srinathan SK, McAuley DF, Mrkobrada M, Levine O, Ribic C (2014). The statistical significance of randomized controlled trial results is frequently fragile: a case for a Fragility Index. J Clin Epidemiol.

[5] Salman RA-S, Beller E, Kagan J, Hemminki E, Phillips RS, Savulescu J, Macleod M, Wisely J, Chalmers I (2014). Increasing value and reducing waste in biomedical research regulation and management. Lancet.

[6] Kearney A, Daykin A, Shaw ARG, Lane AJ, Blazeby JM, Clarke M, Williamson P, Gamble C (2017). Identifying research priorities for effective retention strategies in clinical trials. Trials..

[7] Page SJ, Persch AC (2013). Recruitment, retention, and blinding in clinical trials. Am J Occup Ther.

[8] Brunsdon D, Biesty L, Brocklehurst P, Brueton V, Devane D, Elliott J, Galvin S, Gamble C, Gardner H, Healy P, Hood K, Jordan J, Lanz D, Maeso B, Roberts A, Skene I, Soulsby I, Stewart D, Torgerson D, Treweek S, Whiting C, Wren S, Worrall A, Gillies K (2019). What are the most important unanswered research questions in trial retention? A James Lind Alliance Priority Setting Partnership: the PRioRiTy II (Prioritising Retention in Randomised Trials) study. Trials..

[9] Gillies K, Kearney A, Keenan C, Treweek S, Hudson J, Brueton VC, et al. Strategies to improve retention in randomised trials. Cochrane Database Syst Rev. 2021;3(4). 10.1002/14651858.MR000032.pub3. 10.1002/14651858.MR000032.pub3 PMC8092429 33675536

[10] Tai SS, Nazareth I, Haines A, Jowett C (1997). A randomized trial of the impact of telephone and recorded delivery reminders on the response rate to research questionnaires. J Public Health.

[11] Severi E, Free C, Knight R, Robertson S, Edwards P, Hoile E (2011). Two controlled trials to increase participant retention in a randomized controlled trial of mobile phone-based smoking cessation support in the United Kingdom. Clin Trials.

[12] Free C, Whittaker R, Knight R, Abramsky T, Rodgers A, Roberts IG. Txt2stop: a pilot randomised controlled trial of mobile phone-based smoking cessation support. Tobacco control. 2009;18(2):88-91. 10.1136/tc.2008.026146 19318534

[13] Hoile EC, Free C, Edwards PJ, Felix LM. Methods to increase response rates for data collected by telephone. Cochrane Database of Systematic Reviews. 2009(3). 10.1002/14651858.MR000008.pub4 PMC8941848 19588449

[14] Dierikx TH, Berkhout DJC, Visser L, Benninga MA, Roeselers G, de Boer NKH, et al. The influence of timing of Maternal administration of Antibiotics during cesarean section on the intestinal Microbial colonization in Infants (MAMI-trial): study protocol for a randomised controlled trial. Trials. 2019;20(1):479. 10.1186/s13063-019-3552-8 PMC6683546 31382981

[15] Carter TH, Oliver WM, Graham C, Duckworth AD, White TO. Medial malleolus: Operative Or Non-operative (MOON) trial protocol - a prospective randomised controlled trial of operative versus non-operative management of associated medial malleolus fractures in unstable fractures of the ankle. Trials. 2019;20(1):565. 10.1186/s13063-019-3642-7 PMC6739910 31514744

[16] Crawford P, Thai C, Obholz J, Schievenin J, True M, Shah SA, et al. Assessment of the effeCt of lIfestyle iNtervention plus water-soluble ciNnAMon extract On loweriNg blood glucose in pre-diabetics, a randomized, double-blind, multicenter, placebo controlled trial: study protocol for a randomized controlled trial. Trials. 2016;17(1):1-6. 10.1186/s13063-015-1138-7 PMC4702386 26732017

[17] Treweek S, Bevan S, Bower P, Campbell M, Christie J, Clarke M, et al. Trial forge guidance 1: what is a study within a trial (SWAT)? Trials. 2018;19(1):1-5. 10.1186/s13063-018-2535-5 PMC5824570 29475444

[18] Pirosca S, Shiely F, Clarke M, Treweek S. Tolerating bad health research: the continuing scandal. Trials. 2022. 10.21203/rs.3.rs-1113501/v1. 10.1186/s13063-022-06415-5 PMC9161194 35655288

