# Supplementary material for: How much is the lack of retention evidence costing trial teams in Ireland and the UK?
Source: Trials. 2022 May 12;23:396. doi: 10.1186/s13063-022-06223-x (PMC9097420; doi:10.1186/s13063-022-06223-x)
Supplement: Supplementary file 1 — Additional file 1. [file 13063_2022_6223_MOESM1_ESM.docx]

**Additional File 1**

**Table of Content Page**

Preface and overview of assumptions included in the costing model 2

Assumptions - Hypothetical trial – Ireland 5

Assumptions - Hypothetical trial – United Kingdom 9

Assumptions - MAMI trial 13

Assumptions - MOON trial 18

Assumptions – CINNAMON trial 22

References 27

**Preface**

We acknowledge that the assumptions that we have made regarding the use of the ten retention strategies may not be truly representative of how these activities are or were actually conducted, and that others may make different assumptions.

We have sought input from clinical trial professionals such as trial managers, clinical research nurses, and professionals working in clinical research facilities in the UK and Ireland as well as from the authors of Kearney et al paper (1) for information to inform our assumptions and costing model. Although we have tried to be as realistic and accurate as possible, the overall aim of our project is rather to highlight the lack of evidence to support the use of retention strategies that are being routinely used by CTUs in the UK and that none of these strategies is without cost.

However, to help to address this limitation, we have made the costing spreadsheet available as an additional file, which means readers can modify it to suit their own trial.

**Assumptions about the retention strategies and additional activities that were made regarding the implementations of strategies listed in Kearney et al., (1)**

In the Kearney et al., (1) paper CTUs were able to reply to the survey outlining strategies to mitigate missing data that they routinely use. However responses lacked detail as to how the trial team conducted the activities, which meant we had to make assumptions as to how the top 10 most routinely used retention strategies are likely to be carried out, in discussion with trial managers, research nurses and others.

- “Newsletter” - we calculated the costs of both electronically and manually posting out newsletters.
- “A timeline of participant visits for sites” – the nature of the strategy itself was unclear from the Kearney paper (1), therefore we created two assumptions. Firstly we interpreted this strategy as the Data Management team developing reminder schedule software to send out to each site in the trial. The reminder schedule software can be modified to suit the trial follow-up schedule and used to notify site staff of participants’ visits. Secondly, from correspondents with trial staff we integrated a participant reminder schedule stemming from the site reminder, this involves the Trial Manager/Research Nurse contacting the participants to remind them of their visit and failed contact attempts are also costed. We costed both of these assumptions. The cost of developing and sending site the reminder schedule software was costed separately but also had to be included in the reminder schedule for participants.
- “Inclusion of pre-paid envelopes (questionnaires)” – We costed sending out pre-paid return envelopes with a questionnaire to all participants. We decided to include the costs of a pre-paid return reminder schedule for the questionnaires that are sent out. This reminder schedule is sent to 30% of participants (non-response rate of 30%).
- “Telephone reminders” - we included costs for telephone reminders for trial visits, telephone reminders for questionnaire response for 30% of trial participants and additionally for one of the “real-life” trials (MAMI trial)(2) we included telephone reminders for at home data collection for 30% of trial participants. We also included costs for failed telephone reminder attempts for trial visits, for questionnaire response and additionally for the MAMI trial we included failed telephone reminder attempts for at home data collection, as it is likely multiple attempts may be needed to contact non-responding participants.
- “Data collection scheduled with routine care” is costed as the time it takes for research nurses to collected trial related data from participants during their routine care visit.
- “Site initiation training on missing data”/ “Investigator meetings face-to-face”/ “Routine site visits by CTU staff” – we made the same assumption across all three of these retention strategies – based on input from clinical trial professionals in Ireland these activities are charged at a flat rate plus travel costs and other expenses. In the UK the Trial Manager is paid per hours work plus travel costs and other expenses.
- With regards to “targeted recruitment of GPs/site” there was no clear explanation as to what this entailed outlined in the Kearney et al., paper (1), therefore we interpreted this as trial teams targeting sites that would be most likely to participate/sites that had the facilities and experience to participate/sites that have conducted similar trials previously, we costed the time taken to conduct background research into which sites these would be and associated investigation into recruitment.
- Flexibility in appointments – we made the assumption that only 10% of patients need out of hours appointments which would take 1.5 hours to conduct.

Further detail on the assumptions for each retention strategy in each trial is outlined below

**Mailchimp electronic newsletter costs**

- Based on the standard package for up to 500 recipients the cost is $14.99 per month which works out as $179.88 per year. Based on the conversion rates of 1 GBP = 1.16279 EUR,
- 1 EUR = 0.860001 GBP, 1 USD = 0.843802 EUR and 1 USD = 0.725647 GBP,
- taken from [Xe Currency Converter - LIVE Foreign Exchange Rates](https://www.xe.com/currencyconverter/) on the 7^th^ of September 2021.
- This works out as €151.78 per year and £130.53 per year.

Tables display the trial information and strategy assumptions applied to each of the trials used in the study. The “real-life” trials were sourced out of convenience from [Trials | Home page (biomedcentral.com)](https://trialsjournal.biomedcentral.com/) published between 2016 and 2020.

**Hypothetical trial – Ireland**

| **Trial Characteristics** | |
| --- | --- |
| **Number of participants** | 500 |
| **Number of sites** | 10 sites |
| **Duration of participant follow-up** | 1 year |
| **Number of trial visits** | 3 |
| **Data collection** | 1 questionnaire is sent out and 3 trial visits |
| **Location of the trial** | Ireland |

**Assumptions for costing application**

**Staff costs:** Staff hourly salary was obtained from personal correspondence with Research Nurses and other professionals involved in the conduct and running of clinical trials in Ireland**.**

Research Nurse: €54 hourly

Research Assistant: €25 hourly

Data Manager: €25 hourly

**Stamp cost:** €1.10 [E00127369-GuidePostalRatesA5-Final-AW_V4-2020_WEB.pdf (anpost.com)](https://www.anpost.com/AnPost/media/PDFs/Postal%20Rates/E00127369-GuidePostalRatesA5-Final-AW_V4-2020_WEB.pdf)

**Strategy assumptions**

| **Strategy** | **Assumptions made** |
| --- | --- |
| **Newsletter** | - 2 newsletters sent out over the 1-year trial period - 5 hours to develop the newsletter. - 2 hours work to electronically send out 500 newsletters (this includes time to deal with returned undelivered emails and re-sending) - 8 hours to manually post out 500 newsletters - Stamp costs in Ireland = €1.10 - Mailchimp subscription is €151.78 for 1 year - Developing the newsletter carried out by a Research Nurse. - Emailing/posting out the newsletter carried out by a Research Assistant. |
| **A timeline of participant visits for sites** | - **Reminder schedule for site** - Data Manager develops reminder schedule software - 3 hours of work - Electronically emails the reminder schedule software to the sites - 10 minutes work (0.167 hours) - Carried out once - This reminder schedule software can be modified to suit the trial follow-up schedule and used by trial sites to notify staff of upcoming participant trial visits. - **Built in reminder schedule for participants (this will include the cost of the site reminder schedule)** - Research Nurse contacts the participants directly – telephone call - 5 minutes/0.08hours per call per participant (500 participants) - Carried out three times (once for each visit)   **Failed contact - telephone reminder attempts for *trial visits***   - Carried out by a Research Nurse - An average of 2 failed attempts are made to try contact each participant - Each attempt takes 1 minute so a total time of 2 minutes/0.033hrs per participant (500 participants) - Carried out three times (once for each trial visit) |
| **Inclusion of pre-paid envelopes (questionnaires)** | **Inclusion of pre-paid envelopes**   - This activity is carried out once - Carried out by a Research Assistant - 8 hours work to manually stuff 500 envelopes. - Stamp cost €1.10 - stamp costs are doubled; one stamp for sending out the questionnaire and one stamp for the participant to return the questionnaire   **Reminder schedule**   - This activity is carried out once. - Carried out by a Research Assistant. - Initial response rate estimated at 70%. - Send out reminder schedule of 150 envelopes (30% non-response) - 2.4 hours to manually stuff 150 envelopes (30% of 500) - Stamp costs €1.10 - stamp costs are doubled; one stamp for sending out the questionnaire and one stamp for the participant to return the questionnaire |
| **Telephone reminders** | **Telephone reminders for *trial visits***   - Research Nurse contacts the participants directly - 5 minutes/0.08hours per call per participant (500 participants) - Carried out three times (once for each visit)   **Failed telephone reminder attempts for *trial visits***   - Carried out by a Research Nurse - An average of 2 failed attempts are made to try contact each participant - Each attempt takes 1 minute so a total time of 2 minutes/0.033hrs per participant (500 participants) - Carried out three times (once for each trial visit)   **Telephone reminder schedule for *questionnaire response***   - Carried out once - Carried out by a Research Assistant - Calling up 150 participants (30% non-responders) - 5 minutes/0.08hours per call   **Failed attempts at a telephone reminder for *questionnaire response***   - Carried out once - Carried out by a Research Assistant - 150 participants (30% non-responders) contacted - An average of 2 failed call attempts per participant - Each failed call attempt lasts 1 minute – so total time is 2 minutes/0.033 hours |
| **Data collection scheduled with routine care** | - Carried out by a Research Nurse. - Data is collected during a routine clinic visit. - Portion of routine visit spent on trial data collection: 15 minutes/0.25hrs - Activity carried out three times (once for each trial visit) |
| **Site initiation training on missing data** | - This activity is carried out once for each site (10 sites)   Assume: Training preparation, site training and report per site @€400 per day plus travel and overnight costs.   - Cost per site is €400 which includes staff costs - Travel costs: €100 - Other expenses: overnight costs: €200 |
| **Investigator meetings face-to-face** | - This activity is carried out twice for each site (10 sites)   Assume: Visit preparation, face-to-face meeting and report per site @€400 per day plus travel and overnight costs.   - Cost per site is €400 which includes staff costs - Travel costs: €100 - Other expenses: overnight costs: €200 |
| **Routine site visits by CTU staff** | - This activity is carried out twice for each site (10 sites)   Assume: Visit preparation, site visit and report per site @€400 per day plus travel and overnight costs.   - Cost per site is €400 which includes staff costs - Travel costs: €100 - Other expenses: overnight costs: €200 |
| **Targeted recruitment of GPs/sites** | - Site selection and associated investigation into recruitment (1 hour work per site) - Assumed that 30 sites were targeted to recruit 10 sites (Recruitment to targeting ratio is 1:3) - Carried out by a Research Nurse |
| **Flexibility in appointments** | - Carried out by a Research Nurse. - Length of appointment 1.5 hours - Carried out for a maximum of 10% of the study population which is 50 participants |

**Hypothetical trial – United Kingdom**

| **Trial Characteristics** | |
| --- | --- |
| **Number of participants** | 500 |
| **Number of sites** | 10 sites |
| **Duration of participant follow-up** | 1 year |
| **Number of trial visits** | 3 |
| **Data collection** | 1 questionnaire is sent out and 3 trial visits |
| **Location of the trial** | United Kingdom |

**Assumptions for costing application**

**Staff costs:** Staff hourly salary was obtained from personal correspondence with Research Nurses and other professionals involved in the conduct and running of clinical trials in the United Kingdom.

Trial Manager: £30.40 hourly

Research Nurse: £36 hourly

Research Assistant: £28.85 hourly

Data Manager: £30.40 hourly

**Stamp costs:** £0.66 [Stamps | Royal Mail](https://www.royalmail.com/sending/stamps)

**Strategy Assumptions**

| **Strategy** | **Assumptions made** |
| --- | --- |
| **Newsletter** | - 2 newsletters sent out over the 1-year trial period - 5 hours to develop the newsletter. - 2 hours work to electronically send out 500 newsletters (this includes time to deal with returned undelivered emails and re-sending) - 8 hours to manually post out 500 newsletters - Second class mail – 66p - Mailchimp subscription cost £130.53 for 1 year. - Developing the newsletter is carried out by a Trial Manager. - Emailing/Posting out the newsletter carried out by a Research Assistant. |
| **A timeline of participant visits for sites** | - **Reminder schedule for site** - Data Manager develops reminder schedule software - 3 hours of work - Electronically emails the reminder schedule software to the sites - 10 minutes work (0.167 hours) - Carried out once - This reminder schedule software can be modified to suit the trial follow-up schedule and used by trial sites to notify staff of upcoming participant trial visits - **Built in reminder schedule for participants (this will include the cost of the site reminder schedule)** - Trial Manager contacts the participants directly - 5 minutes/0.08hours per call per participant (500 participants) - Carried out three times (once for each trial visit)   **Failed contact - telephone reminder attempts for *trial visits***   - Carried out by a Trial Manager - An average of 2 failed attempts are made to try contact each participant - Each attempt takes 1 minute so a total time of 2 minutes/0.033 hours per participant (500 participants) - Carried out three times (once for each trial visit) |
| **Inclusion of pre-paid envelopes (questionnaires)** | **Inclusion of pre-paid envelopes**   - This activity is carried out once - Carried out by a Research Assistant - 8 hours work to manually stuff 500 envelopes. - Stamp costs = 0.66p - stamp costs are doubled; one stamp for sending out the questionnaire and one stamp for the participant to return the questionnaire   **Reminder Schedule**   - Reminder schedule sent out once - Carried out by a Research Assistant - Initial response rate estimated at 70%. - Send out reminder schedule to 150 participants (30% non-response) - 2.4 hours work to manually stuff 150 envelopes - Stamp costs = 0.66p - stamp costs are doubled; one stamp for sending out the questionnaire and one stamp for the participant to return the questionnaire |
| **Telephone reminders** | **Telephone reminders for *trial visits***   - Trial Manager contacts the participants directly - 5 minutes/0.08hours per call per participant (500 participants) - Carried out three times (once for each trial visit)   **Failed telephone reminder attempts for *trial visits***   - Carried out by a Trial Manager - An average of 2 failed attempts are made to try contact each participant - Each attempt takes 1 minute so a total time of 2 minutes/0.033 hours per participant (500 participants) - Carried out three times (once for each trial visit)   **Telephone reminder schedule for *questionnaire response***   - Carried out once. - Carried out by a Research Assistant. - Initial response rate estimated 70% - Telephone reminder schedule for 150 participants (30% non-response), - 5 minutes/0.08hrs per call   **Failed telephone reminder attempts for *questionnaire response***   - Carried out once - Carried out by a Research Assistant - 150 participants (30% non-responders) contacted - An average of 2 failed attempts are made to try contact each participant - Each attempt takes 1 minute so a total time of 2 minutes/0.033hrs per participant. |
| **Data collection scheduled with routine care** | - Carried out by a Research Nurse. - Data is collected during a routine clinic visit. - Portion of routine visit spent on trial data collection: 15 minutes/0.25hrs - Activity is carried out three times (once for each trial visit) |
| **Site initiation training on missing data** | - Trial Manager carries out preparation, carries out training and writes up a report - 11 hours of work in total - Hourly salary for a Trial Manager - Travel costs: £100 - Other expenses: overnight costs: £200 - Activity carried out once per site (10 sites) |
| **Investigator meetings face-to-face** | - Trial Manager carries out preparation, carries out face-to-face meeting and writes up a report - 11 hours of work in total - Hourly salary for a Trial Manager - Travel costs: £100 - Other expenses: overnight costs: £200 - Activity carried out twice per site (10 sites) |
| **Routine site visits by CTU staff** | - Trial Manager carries out preparation, carries out visit and writes up a report - 11 hours of work in total - Hourly salary for a Trial Manager - Travel costs: £100 - Other expenses: overnight costs: £200 - Activity carried out twice per site (10 sites) |
| **Targeted recruitment of GPs/sites** | - Site selection and associated investigation into recruitment (1 hour work per site). - Assumed that 30 sites are targeted to recruit 10 sites (Recruitment to targeting ratio is 1:3) - Carried out by a Trial Manager |
| **Flexibility in appointments** | - Carried out by a Research Nurse. - Length of appointment 1.5 hours - Carried out for a maximum of 10% of the study population which is 50 participants. |

**MAMI trial** (2) The influence of timing of Maternal administration of Antibiotics during cesarean section on the intestinal Microbial colonization in Infants (MAMI-trial): study protocol for a randomised controlled trial.

**Trial information**

| **Trial Characteristics** | |
| --- | --- |
| **Number of participants** | 60 |
| **Number of sites** | 1 site (single centre) |
| **Duration of participant follow-up** | 2 years |
| **Number of trial visits** | 1 |
| **Data collection** | Data collection takes place at an outpatient clinic prior to the birth of the child, takes places at the birth, and at home data collection takes place on day 1 (after birth), day 7, day 28 and when the child is 2 years old  1 postal questionnaire is sent out |
| **Location of the trial** | Amsterdam |

**Assumptions for costing application**

**Staff costs –** estimates of average staff costs for a Research Nurse, Research Assistant and a Data Manager in the Netherlands - <https://www.salaryexpert.com/salary/job/research-assistant/netherlands> <https://www.salaryexpert.com/salary/job/clinical-research-nurse/netherlands>

[Manager Clinical Data Salary Netherlands - SalaryExpert](https://www.salaryexpert.com/salary/job/manager-clinical-data/netherlands)

Research Nurse: €30 hourly

Research Assistant €17 hourly

Data Manager: €32 hourly

**Stamp costs -** €0.96c **-** [Domestic Mail | PostNL](https://www.postnl.nl/en/sending/letter-or-card/domestic-mail/)

**Strategy Assumptions**

| **Strategy** | **Assumptions made** |
| --- | --- |
| **Newsletter** | - 4 newsletters sent out over the 2-year trial period - 5 hours to develop the newsletter. - 0.24 hours to electronically send out 60 newsletters (2 hours work to electronically send out 500 newsletters, therefore 60 newsletters take 0.24 hours) - 0.96 hours of work to manually stuff and post 60 newsletters (8 hours to manually post out 500 newsletters, therefore 60 newsletters take 0.96 hours) - Stamp costs in the Netherlands = €0.96 - Mailchimp subscription for two years is €303.56 (€151.78 for 1 year) - Developing the newsletter carried out by a Research Nurse. - Emailing/posting out the newsletter carried out by a Research Assistant |
| **A timeline of participant visits for sites** | - **Reminder schedule for site** - Data Manager develops reminder schedule software - 3 hours of work - Electronically emails the reminder schedule software to the sites - 10 minutes work (0.167 hours) - Carried out once - This reminder schedule software can be modified to suit the trial follow-up schedule and used by trial sites to notify staff of upcoming participant trial visits - **Built in reminder schedule for participants (this will include the cost of the site reminder schedule)** - Carried out once (1 trial visit) - Research Nurse contacts the participants directly - 5 minutes/0.08hours per call per participant (60 participants)   **Failed telephone reminder attempts for *trial visits***   - Carried out once (1 trial visit) - Carried out by a Research Nurse - An average of 2 failed attempts are made to try contact each participant - Each attempt takes 1 minute so a total time of 2 minutes/0.033 hours per participant (60 participants) |
| **Inclusion of pre-paid envelopes (questionnaires)** | **Inclusion of pre-paid envelopes**   - This activity is carried out once - Carried out by a Research Assistant - 0.96 hours work to manually stuff 60 envelopes. - Stamp cost €0.96 - stamp costs are doubled; one stamp for sending out the questionnaire and one stamp for the participant to return the questionnaire   **Reminder schedule**   - This activity is carried out once. - Carried out by a Research Assistant. - Initial response rate estimated at 70%. - Send out reminder schedule to 18 participants (30% non-response) - 0.288 hours to manually stuff 18 envelopes (30% non-response). - Stamp costs €0.96 - stamp costs are doubled; one stamp for sending out the questionnaire and one stamp for the participant to return the questionnaire |
| **Telephone reminders** | **Telephone reminders for *trial visits***   - Carried out once (1 trial visit) - Research Nurse contacts the participants directly - 5 minutes/0.08hours per call per participant (60 participants)   **Failed telephone reminder attempts for *trial visits***   - Carried out once (1 trial visit) - Carried out by a Research Nurse - An average of 2 failed attempts are made to try contact each participant - Each attempt takes 1 minute so a total time of 2 minutes/0.033 hours per participant (60 participants)   **Telephone reminder schedule for *questionnaire response***   - Carried out once - Carried out by a Research Assistant - Calling up 18 (30% non-responders) participants - 5 minutes/0.08 hours per call   **Failed attempts at a telephone reminder for *questionnaire response***   - Carried out once - Carried out by a Research Assistant - 18 (30% non-responders) participants for the telephone reminder for questionnaire response - An average of 2 failed call attempts per participant - Each failed call attempt lasts 1 minute – so total time is 2 minutes/0.033 hours   **Telephone reminder for at home data collection**   - Carried out 4 times - Carried out by a Research Nurse - Calling up 18 participants (30% non-response) - 1 telephone reminder for each at home data collection event (day 1, 7, 28, 2 years old, 4 in total) - Each call last 5 minutes/0.08 hours per participant   **Failed attempts at telephone reminder calls**   - Activity is carried out 4 times - Carried out by a Research Nurse - 18 participants (30% non-response) - An average of 2 failed call attempts per participant - Each failed call attempt lasts 1 minute – so total time is 2 minutes/0.033 hours per participant |
| **Data collection scheduled with routine care** | - Carried out by a Research Nurse. - Data is collected during a routine clinic visit. - Portion of routine visit spent on trial data collection: 15 minutes/0.25hrs. - Activity carried out twice (outpatient clinic data collection and data collection at birth) |
| **Site initiation training on missing data** | - This activity is carried out once for each site (1 site)   Assume: Training preparation, site training and report per site @€400 per day plus travel and overnight costs.   - Cost per site is €400 which includes staff costs - No travel costs because it is a single centre trial. - Other expenses: No overnight costs because it is a single centre trial |
| **Investigator meetings face-to-face** | - Activity is carried out four times over a 2-year period for each site (1 site)   Assume: Visit preparation, face-to-face meeting, and report per site @€400 per day plus travel and overnight costs.   - Cost per site is €400 which includes staff costs - No travel costs because it is a single centre trial. - Other expenses: No overnight costs because it is a single centre trial. |
| **Routine site visits by CTU staff** | - Activity is carried out four times over a 2-year period for each site (1 site)   Assume: Visit preparation, site visit, and report per site @€400 per day plus travel and overnight costs.   - Cost per site is €400 which includes staff costs - No travel costs because it is a single centre trial. - Other expenses: No overnight costs because it is a single centre trial. |
| **Targeted recruitment of GPs/sites** | - Site selection and associated investigation into recruitment (1 hour work per site) - Single centre study so assumed only 1 site was targeted for recruitment - Carried out by a Research Nurse |
| **Flexibility in appointments** | - Carried out by a Research Nurse. - Length of appointment 1.5 hours - Carried out for a maximum of 10% of the study population which is 6 participants |

**MOON trial** (3) Medial malleolus: Operative Or Non-operative (MOON) trial protocol - a prospective randomised controlled trial of operative versus non-operative management of associated medial malleolus fractures in unstable fractures of the ankle.

**Trial information**

| **Trial Characteristics** | |
| --- | --- |
| **Number of participants** | 154 |
| **Number of sites** | 1 site |
| **Duration of participant follow-up** | 1 year |
| **Number of trial visits** | Outcome assessment at 2 weeks, 6 weeks and 1 year post intervention (3 trial visits) |
| **Data collection** | Postal questionnaires sent out a 3- and 6-month time marks (2 questionnaires) |
| **Location of the trial** | UK based trial |

**Staff costs:** Staff hourly salary was obtained from personal correspondence with Research Nurses and other professionals involved in the conduct and running of clinical trials in the United Kingdom.

Trial Manager: £30.40 hourly

Research Nurse: £36 hourly

Research Assistant: £28.85 hourly

Data Manager: £30.40 hourly

**Stamp costs:** £0.66 [Stamps | Royal Mail](https://www.royalmail.com/sending/stamps)

**Strategy Assumptions**

| **Strategy** | **Assumptions made** |
| --- | --- |
| **Newsletter** | - 2 newsletters are sent over a 1-year trial. - 5 hours to develop the newsletter. - 0.616 hours to electronically send out 154 newsletters (2 hours work to electronically send out 500 newsletters, therefore 154 newsletters is 0.616 hours work) - 2.464 hours to manually post out 154 newsletters (8 hours to manually post out 500 newsletters, therefore 154 newsletters take 2.464 hours) - Second class mail – 66p - Mailchimp subscription cost £130.53 for 1 year. - Developing the newsletter is carried out by a Trial Manager. - Emailing/Posting out the newsletter carried out by a Research Assistant. |
| **A timeline of participant visits for sites** | - **Reminder schedule for site** - Data Manager develops reminder schedule software - 3 hours of work - Electronically emails the reminder schedule software to the sites - 10 minutes work (0.167 hours) - Carried out once - This reminder schedule software can be modified to suit the trial follow-up schedule and used by trial sites to notify staff of upcoming participant trial visits - **Built in reminder schedule for participants (this will include the cost of the site reminder schedule)** - Trial Manager contacts the participants directly - 5 minutes/0.08hours per call per participant (154 participants) - Carried out three times (once for each trial visit)   **Failed contact - telephone reminder attempts for *trial visits***   - Carried out three times (once for each trial visit) - Carried out by a Trial Manager - An average of 2 failed attempts are made to try contact each participant (154 participants) - Each attempt takes 1 minute so a total time of 2 minutes/0.033hours per participant |
| **Inclusion of pre-paid envelopes (questionnaires)** | **Inclusion of pre-paid envelopes**   - This activity is carried out twice (2 questionnaires are posted out in this study) - Carried out by a Research Assistant. - 2.464 hours to manually stuff 154 envelopes - Stamp costs = 0.66p - stamp costs are doubled; one stamp for sending out the questionnaire and one stamp for the participant to return the questionnaire   **Reminder Schedule**   - Reminder schedule sent out twice (once for each questionnaire) - Carried out by a Research Assistant - Initial response rate estimated at 70%. - Send out reminder schedule of 47 envelopes (30% non-response) - 0.752 hours of work to manually stuff 47 envelopes - Stamp costs = 0.66p - stamp costs are doubled; one stamp for sending out the questionnaire and one stamp for the participant to return the questionnaire |
| **Telephone reminders** | **Telephone reminders for *trial visits***   - Trial Manager contacts the participants directly - 5 minutes/0.08hours per call per participant (154 participants) - Carried out three times (once for each trial visit)   **Failed telephone reminder attempts for *trial visits***   - Carried out three times (once for each trial visit) - Carried out by a Trial Manager - An average of 2 failed attempts are made to try contact each participant (154 participants) - Each attempt takes 1 minute so a total time of 2 minutes/0.033hours per participant   **Telephone reminder schedule for *questionnaire response***   - Carried out twice (once for each questionnaire) - Carried out by a Research Assistant. - Initial response rate estimated 70% - Telephone reminder schedule for 47 participants (30% non-response), - 5 minutes/0.08hours per call per participant   **Failed telephone reminder attempts for *questionnaire response***   - Carried out twice (once for each questionnaire) - Carried out by a Research Assistant - An average of 2 failed attempts are made to try contact each participant (47 participants) - Each attempt takes 1 minute so a total time of 2 minutes/0.033hours per participant. |
| **Data collection scheduled with routine care** | **Data collected scheduled with routine care**   - Carried out by a Research Nurse. - Data is collected during a routine clinic visit. - Portion of routine visit spent on trial data collection: 15 minutes/0.25hrs - Activity is carried out 3 times (for each trial visit) |
| **Site initiation training on missing data** | - Trial Manager carries out preparation, carries out training and writes up a report - 11 hours of work in total - Hourly salary for a Trial Manager - No travel costs since it is a single centre trial. - Other expenses: No overnight costs since it is a single centre trial - Activity carried out once per site (1 site) |
| **Investigator meetings face-to-face** | - Trial Manager carries out preparation, carries out face-to-face meeting and writes up a report - 11 hours of work in total - Hourly salary for a Trial Manager - No travel costs since it is a single centre trial. - Other expenses: No overnight costs because it is a single centre trial - Activity carried out twice per site (1 site) |
| **Routine site visits by CTU staff** | - Trial Manager carries out preparation, carries out visit and writes up a report - 11 hours of work in total - Hourly salary for a Trial Manager - No travel costs since it is a single centre study. - Other expenses: No overnight costs because it is a single centre trial - Activity carried out twice per site (1 site) |
| **Targeted recruitment of GPs/sites** | - Site selection and associated investigation into recruitment (1 hour work per site). - Single centre study so assumed only 1 site was targeted for recruitment - Carried out by a Trial Manager |
| **Flexibility in appointments** | - Carried out by a Research Nurse. - Length of appointment 1.5 hours - Carried out for a maximum of 10% of the study population which is 16 participants. |

**CINNAMON trial** (4) Assessment of the effeCt of lIfestyle iNtervention plus water-soluble ciNnAMon extract On loweriNg blood glucose in pre-diabetics, a randomized, double-blind, multicenter, placebo controlled trial: study protocol for a randomized controlled trial.

**Trial information**

| **Trial Characteristics** | |
| --- | --- |
| **Number of participants** | 428 |
| **Number of sites** | 5 sites |
| **Duration of participant follow-up** | 2-years |
| **Number of trial visits** | 9 trial visits |
| **Data collection** | Data collection takes place at each trial visit (9 visits) |
| **Location of the trial** | USA based trial |

**Staff costs** - estimates of average staff costs for both a Research Nurse and a Research Assistant in the Netherlands - <https://www.salaryexpert.com/salary/job/clinical-research-nurse/united-states>

<https://www.salaryexpert.com/salary/job/research-assistant/united-states>

[Manager Clinical Data Salary the United States - SalaryExpert](https://www.salaryexpert.com/salary/job/manager-clinical-data/united-states)

Research Nurse: $40 hourly

Research Assistant: $20 hourly

Data Manager: $42 hourly

**Stamp costs:** 0.55$ - [Stamps.com - Current Postage Rates, US Postage Stamp Prices](https://www.stamps.com/usps/current-postage-rates/)

**Strategy Assumptions**

| **Strategy** | **Assumptions made** |
| --- | --- |
| **Newsletter** | - 4 newsletters are sent over a 2-year trial period. - 5 hours to develop the newsletter. - 1.712 hours work to electronically send out 428 newsletters (2 hours work to electronically send out 500 newsletters, therefore 428 newsletters take 1.712 hours work) - 6.848 hours to manually post out 428 newsletters (8 hours to manually post out 500 newsletters, therefore 428 newsletters take 6.848 hours) - Postage costs = 0.55$ - Mailchimp subscription cost $179.88 for 1 year, so two-year subscription is $359.76 - Developing the newsletter is carried out by a Research Nurse. - Emailing/Posting out the newsletter carried out by a Research Assistant. |
| **A timeline of participant visits for sites** | - **Reminder schedule for site** - Data Manager develops reminder schedule software - 3 hours of work - Electronically emails the reminder schedule software to the sites - 10 minutes work (0.167 hours) - Carried out once - This reminder schedule software can be modified to suit the trial follow-up schedule and used by trial sites to notify staff of upcoming participant trial visits - **Built in reminder schedule for participants (this will include the cost of the site reminder schedule)** - Carried out 9 times (once for each trial visit) - Research Nurse contacts the participants directly - 5 minutes/0.08hours per call per participant. - 428 participants   **Failed contact - telephone reminder attempts for *trial visits***   - Carried out 9 times (once for each trial visit) - Carried out by a Research Nurse - An average of 2 failed attempts are made to try contact each participant - Each attempt takes 1 minute so a total time of 2 minutes/0.033 hours per participant (428 participants) |
| **Inclusion of pre-paid envelopes (questionnaires)** | **Inclusion of pre-paid envelopes**   - This activity is carried out once. - Carried out by a Research Assistant. - 6.848 hours to manually stuff 428 envelopes - Stamp costs = 0.55$ - stamp costs are doubled; one stamp for sending out the questionnaire and one stamp for the participant to return the questionnaire   **Reminder Schedule**   - Carried out by a Research Assistant - Reminder schedule sent out once - Initial response rate estimated at 70%. - Send out reminder schedule to 129 participants (30% non-response) - 2.064 hours of work - Stamp costs = 0.55$ - stamp costs are doubled; one stamp for sending out the questionnaire and one stamp for the participant to return the questionnaire |
| **Telephone reminders** | **Telephone reminders for *trial visits***   - Carried out 9 times (once for each trial visit) - Research Nurse contacts the participants directly - 5 minutes/0.08hours per call per participant. - 428 participants   **Failed telephone reminder attempts for *trial visits***   - Carried out 9 times (once for each trial visit) - Carried out by a Research Nurse - An average of 2 failed attempts are made to try contact each participant - Each attempt takes 1 minute so a total time of 2 minutes/0.033 hours per participant (428 participants)   **Telephone reminder schedule for *questionnaire response***   - Carried out once - Carried out by a Research Assistant. - Initial response rate estimated 70% - Telephone reminder schedule for 129 participants (30% non-response), - 5 minutes/0.08 hours per call per participant   **Failed telephone reminder attempts for *questionnaire response***   - Carried out once - Carried out by a Research Assistant - An average of 2 failed attempts are made to try contact each participant (129 participants) - Each attempt takes 1 minute so a total time of 2 minutes/0.033 hours per participant |
| **Data collection scheduled with routine care** | **Data collection scheduled with routine care**   - Carried out by a Research Nurse. - Data is collected during a routine clinic visit. - Portion of routine visit spent on trial data collection: 15 minutes/0.25hrs - Activity is carried out 9 times (for each trial visit) |
| **Site initiation training on missing data** | - Activity is carried out once per site (5 sites)   Assume: Training preparation, site training and report per site @€400 per day plus travel and overnight costs.   - Cost per site is $400 which includes staff costs - Travel $100 average estimate. - Other expenses: Overnight costs $200 average estimate. |
| **Investigator meetings face-to-face** | - Activity is carried out four times over a 2-year period for each site (5 sites)   Assume: Visit preparation, face-to-face meeting, and report per site @€400 per day + travel + overnight.   - Cost per site is $400 which includes staff costs - Travel $100 average estimate. - Other expenses: Overnight costs $200 average estimate. |
| **Routine site visits by CTU staff** | - Activity is carried out four times over a 2-year period for each site (5 sites)   Assume: Visit preparation, site visit and report per site @€400 per day + travel + overnight Cost per site is $400 which includes staff costs   - Travel $100 average estimate. - Other expenses: Overnight costs $200 average estimate. |
| **Targeted recruitment of GPs/sites** | - Site selection and associated investigation into recruitment (1 hour work per site). - 5 sites were recruited so assumed 15 sites were initially targeted for recruitment (Recruitment to targeting ratio is 1:3) - Carried out by a Research Nurse |
| **Flexibility in appointments** | - Carried out by a Research Nurse. - Length of appointment 1.5 hours - Carried out for a maximum of 10% of the study population which is 43 participants. |

*References*

1. Kearney A, Daykin A, Shaw ARG, Lane AJ, Blazeby JM, Clarke M, et al. Identifying research priorities for effective retention strategies in clinical trials. Trials. 2017;18(1):406.

2. Dierikx TH, Berkhout DJC, Visser L, Benninga MA, Roeselers G, de Boer NKH, et al. The influence of timing of Maternal administration of Antibiotics during cesarean section on the intestinal Microbial colonization in Infants (MAMI-trial): study protocol for a randomised controlled trial. Trials. 2019;20(1):479.

3. Carter TH, Oliver WM, Graham C, Duckworth AD, White TO. Medial malleolus: Operative Or Non-operative (MOON) trial protocol - a prospective randomised controlled trial of operative versus non-operative management of associated medial malleolus fractures in unstable fractures of the ankle. Trials. 2019;20(1):565.

4. Crawford P, Thai C, Obholz J, Schievenin J, True M, Shah SA, et al. Assessment of the effeCt of lIfestyle iNtervention plus water-soluble ciNnAMon extract On loweriNg blood glucose in pre-diabetics, a randomized, double-blind, multicenter, placebo controlled trial: study protocol for a randomized controlled trial. Trials. 2016;17(1):1-6.
